# Achieving High Thermal Conductivity and Satisfactory Insulating Properties of Elastomer Composites by Self-Assembling BN@GO Hybrids

**DOI:** 10.3390/polym15030523

**Published:** 2023-01-19

**Authors:** Xing Xie, Dan Yang

**Affiliations:** 1 College of Materials Science and Engineering, Beijing University of Chemical Technology, Beijing 100029, China; 2 College of New Materials and Chemical Engineering, Beijing Institute of Petrochemical Technology, Beijing 102617, China

**Keywords:** thermal conductivity, insulating properties, surface modification, self-assembling, dielectric

## Abstract

With increasing heat accumulation in advanced modern electronic devices, dielectric materials with high thermal conductivity (λ) and excellent electrical insulation have attracted extensive attention in recent years. Inspired by mussel, hexagonal boron nitride (hBN) and graphene oxide (GO) are assembled to construct mhBN@GO hybrids with the assistance of poly(catechol-polyamine). Then, mhBN@GO hybrids are dispersed in carboxy nitrile rubber (XNBR) latex via emulsion coprecipitation to form elastomer composites with a high λ and satisfactory insulating properties. Thanks to the uniform dispersion of mhBN@GO hybrids, the continuous heat conduction pathways exert a significant effect on enhancing the λ and decreasing the interface thermal resistance of XNBR composites. In particular, the λ value of 30 vol% mhBN@GO/XNBR composite reaches 0.4348 W/(m·K), which is 2.7 times that of the neat XNBR (0.1623 W/(m·K)). Meanwhile, the insulating hBN platelets hinder the electron transfer between adjacent GO sheets, leading to satisfactory electrical insulation in XNBR composites, whose AC conductivity is as low as 10^−10^ S/cm below 100 Hz. This strategy opens up new prospects in the assembly of ceramic and carbonaceous fillers to prepare dielectric elastomer composites with high λ and satisfactory electrical insulation, making them promising for modern electrical systems.

## 1. Introduction

With the high degree of integration and miniaturization of electronic devices, a large amount of heat is accumulated in a small volume of the electronic components in operation. In that regard, efficient heat dissipation has emerged as a critical issue that urgently needs to be addressed to ensure the service life, reliability, and stability of electronic devices [1,2,3,4]. Polymeric composites are widely utilized as thermal management materials in electronic packing and engineering due to their excellent characteristics, including high resistivity, low cost, lightweight, and easy processing [5,6]. Elastomers, in particular, are usually used as thermal pads or thermal greases [6,7,8]. Due to its large amounts of strong polar groups, the carboxy nitrile rubber (XNBR), which shows a relatively high dielectric constant, has been considered as a candidate thermal management material in electron components. Nevertheless, the intrinsic thermal conductivity (λ) of elastomers is usually low (0.16–0.22 W/(m·K)), which does not meet the requirements for heat transfer in advanced electronic components [9,10,11].

In order to increase the λ of polymers, an effective solution consists of incorporating high thermally conductive fillers into the polymeric matrix, such as ceramics, metals, and carbon materials [2,12,13]. Hexagonal boron nitride (hBN) has attracted considerable attention from researchers due to its high λ and excellent electrical insulation. Unsatisfactorily, the pristine hBN is poorly compatible with polymers and exhibits a nonuniform dispersion within the polymeric matrix, thereby restraining the growth of λ in the final composites [14,15]. In recent years, the carbonaceous fillers with high electrical conductivity, such as graphene and graphene oxide (GO), have been shown to be able to increase the λ of polymeric composites at a low loading [16]. Nevertheless, the application of such fillers is unavoidably limited in fields where electrical insulation is required. Therefore, designing and preparing dielectric polymeric composites with high λ and outstanding electrical insulation still remain a challenge [17].

Blocking the electron transmission between neighboring carbonaceous fillers by depositing insulating layers or particles is a reliable way to enhance the λ of the polymeric composites while maintaining their electrical insulating properties [15,18]. Shen et al. [15] utilized silica nanoparticles to coat graphene nanoplatelets (GNPs) to construct Silica@GNPs complexes. Then, the obtained Silica@GNPs complexes were incorporated into a polydimethylsiloxane (PDMS) matrix to fabricate Silica@GNP/PDMS composites whose λ value (0.497 W/(m·K)) was found to be 155% that of the neat PDMS (0.195 W/(m·K)). Nevertheless, the electrical resistivity of PDMS composites was still as high as 10^13^ Ω∙cm, ensuring their excellent insulation. Guo et al. [2] first functionalized Al_2_O_3_ nanoparticles by γ-aminopropyltriethoxysilane (denoted as f-Al_2_O_3_) and then covered them with GO to form f-Al_2_O_3_@RGO hybrids, which were afterward used as thermally conductive fillers for improving the λ of the nanofibrillated cellulose (NFC) matrix. Given the synergistic effect of both Al_2_O_3_ and RGO, the in-plane λ of the as-prepared f-Al_2_O_3_@RGO/NFC composite could be increased to 8.3 W/(m·K) at the RGO and f-Al_2_O_3_ loadings of 30 wt% and 5.6 wt%, respectively. This result was 2075% of the neat NFC (0.4 W/(m·K)). Meanwhile, the measured electrical resistivity of f-Al_2_O_3_@RGO/NFC composites exceeded 10^9^ Ω∙cm, thus meeting the requirements for electrical insulation.

Inspired by marine mussels, Messersmith et al. [19] discovered that dopamine containing catechol and amine functional groups can self-polymerize to form strongly adhesive polydopamine (PDA) in the alkaline buffer aqueous solution. Nevertheless, dopamine is unsuitable for widespread use because of its high cost. Therefore, a cheaper substance as a replacement for the expensive dopamine must be found. Luckily, the inexpensive catechol can react with the economical polyamine to synthesize poly(catechol/polyamine) (PCPA) which exhibits adhesion properties similar to PDA [20]. 

In this work, hBN and GO are assembled to construct mhBN@GO hybrids with the assistance of PCPA to improve the λ of XNBR. Meanwhile, the insulating hBN effectively prevented the connection between adjacent GO sheets, leading to satisfactory electrical insulation for the XNBR composites. The as-prepared XNBR composites might be promising thermal management materials in the future electronic industry. 

## 2. Experimental

### 2.1. Materials

The XNBR latex (Zeon International Trading Co., Ltd., Tokyo, Japan) was chosen as the polymeric matrix. The hBN platelets were obtained from Dandong Rijin Technology Co., Ltd., Dandong, China. The GO sheets were purchased from Chengdu Organic Chemicals Co., Ltd., Chengdu, China. Polyamine (Tetraethylenepentamine, TEPA) and catechol were provided by Bailingwei Technology Co., Ltd., Beijing, China. Dicumyl peroxide (DCP), tris-acid, and other reagents were produced by Maclean’s Reagent Co., Ltd., Beijing, China. 

### 2.2. Preparation Process of mhBN@GO/XNBR Composites

**Figure 1:**
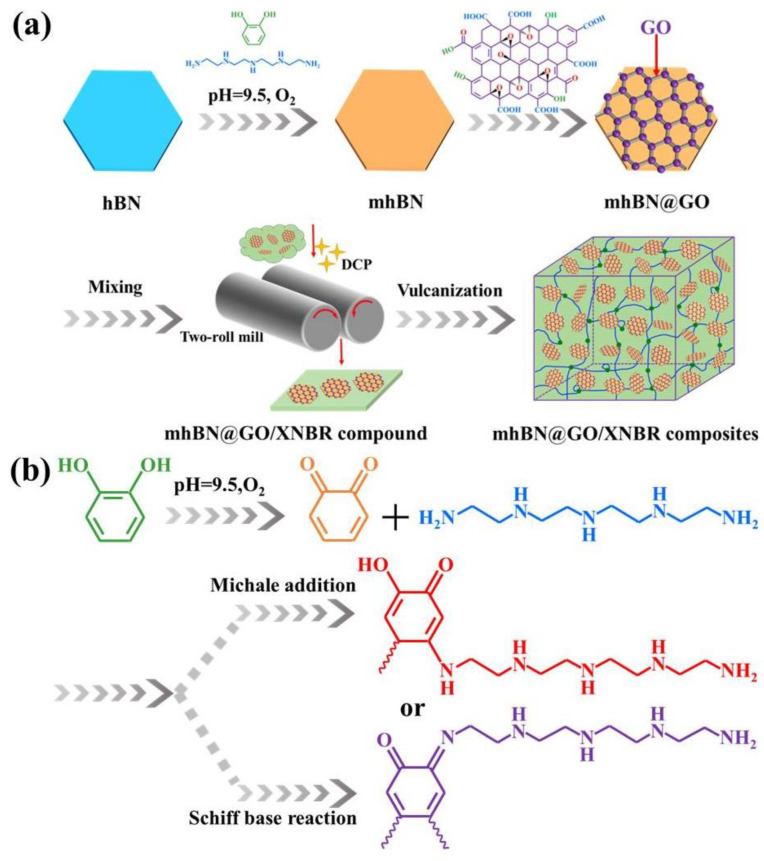
(**a**) Preparation of mhBN@GO hybrids and mhBN@GO/XNBR composites. (**b**) The reaction mechanism between catechol and tetraethylenepentamine.

The fabrication method of mhBN@GO/XNBR composites is illustrated in Figure 1. At the beginning, 5 g of hBN were dispersed in 250 mL of deionized water, and the pH of the mixed solution was adjusted to 9.5 with tris-acid. Then, 0.75 g of catechol and 0.25 g of TEPA were added to the above solution and mechanically stirred at 40 °C for 3 h to obtain the modified hBN hybrids (denoted as mhBN). Subsequently, the GO sheets were dispersed into the blend, which was afterward stirred at 60 °C for 8 h. Finally, the as-obtained mixture was vacuum filtered, washed with deionized water, and vacuum-dried at 60 °C overnight to obtain mhBN@GO hybrids. 

After that, different contents of hBN or mhBN@GO (the volume fractions of 10, 20, and 30 vol%) were incorporated into the XNBR latex via the emulsion coprecipitation method. Initially, the XNBR latex and filler were stirred for 30 min to achieve a uniform dispersion. Then, 1 wt% CaCl_2_ was added as a flocculant to the solution. Next, the mixture was water-washed and vacuum-dried at 60 °C to obtain dry compounds. Finally, the compounds and a certain amount of DCP were blended in a double roll open mixer. After 12 h, all as-prepared compounds were compressed by molding at 160 °C and 12 MPa for their vulcanizing time (T_C90_) to obtain the cured XNBR composites. The T_C90_ values were based on the rheometer (M-3000AU) test data. 

### 2.3. Characterization

The thermogravimetric curves were acquired using a thermogravimetric analysis instrument (TGA, TA SDT650, TA Instruments, New Castle, DE, USA). The surface chemical compositions and surface morphologies of hBN, mhBN, and mhBN@GO were analyzed by means of an X-ray photoelectron spectrometer (XPS, ESCALAB 250, Thermo Electron Corporation, Madison, WI, USA) and a high-resolution transmission electron microscope (HR-TEM, Hitachi H9000, Hitachi Instrument, Tokyo, Japan), respectively. The cross-section morphologies of the XNBR composites were observed by a JSM-5600LV FE-SEM (Thermo Fisher Scientific, Hillsboro, OR, USA). The mechanical properties of the XNBR composites were evaluated using a tensile machine (Instron-3366, Instron Corporation, Norwood, MA, USA). The dielectric behaviors of the composites were studied with a Concept 40 dielectric property tester at room temperature. The λ of the XNBR samples were measured using a flat-panel thermal conductivity meter (DRL-III, Xiangyi Instrument, Xiangtan, China).

## 3. Results and Discussion

Figure 1a shows the preparation process of the mhBN@GO/XNBR composites. The hBN was first modified by PCPA (denoted as mhBN), which was then assembled with GO to synthesize the mhBN@GO hybrids. Next, the as-prepared mhBN@GO was dispersed into the XNBR matrix to obtain mhBN@GO/XNBR composites via a latex mixing method. A possible reaction mechanism for the PCPA has been added to Figure 1b. First, the catechol was oxidized into quinoid structures in an alkalescent tris-acid buffer solution, and then the generated quinoid structures react with polyamine through Michael addition or Schiff base reactions to form an intermolecularly cross-linked PCPA network, which is finally deposited on the surfaces of the hBN platelets [21].

**Figure 2:**
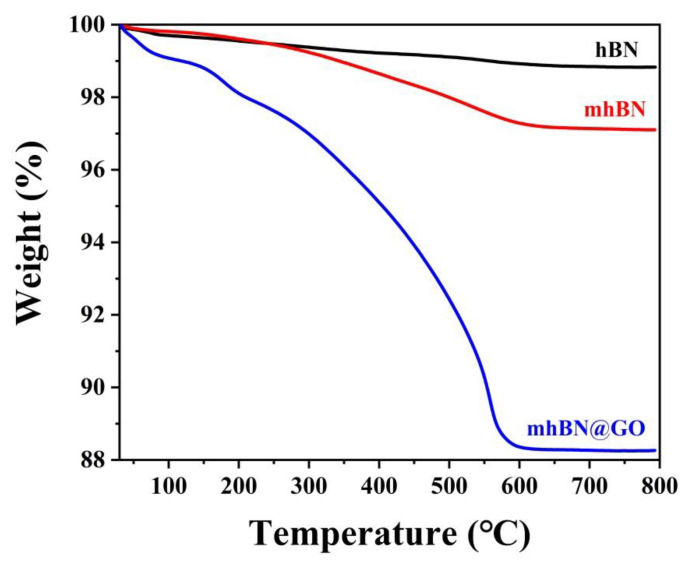
TGA curves of hBN, mhBN, and mhBN@GO.

The TGA curves of hBN, mhBN, and mhBN@GO are shown in Figure 2. It can be seen that the hBN displayed a small weight loss in the range of 30–800 °C, which is ascribed to the excellent thermal stability of hBN. However, a slight weight loss of 2.71% was observed in the TGA curve of mhBN at 600 °C. The larger weight loss of mhBN compared with hBN might be attributed to the degradation of the PCPA layer at the high temperature. In addition, there was also a significant weight loss of 11.64% in the TGA curve of mhBN@GO within the range of 30–600 °C. The increased weight loss of mhBN@GO compared with mhBN was due to the pyrolysis of unstable oxygen-containing groups in GO [22]. 

**Figure 3:**
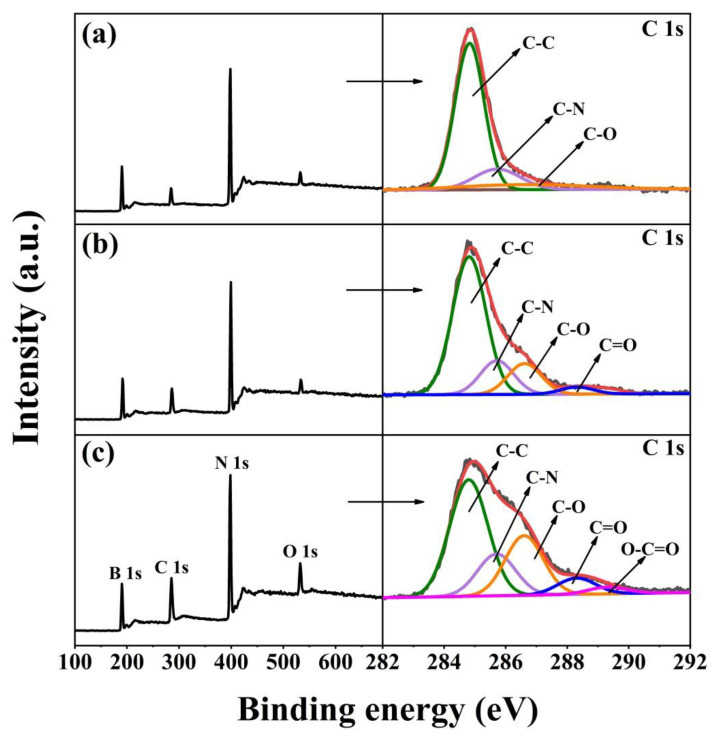
XPS spectra and decomposed C 1s spectra of (**a**) hBN, (**b**) mhBN, and (**c**) mhBN@GO.

The surface chemical elements of hBN, mhBN, and mhBN@GO are analyzed via XPS, and the results are shown in Figure 3 and Table 1. The clearly distinguishable peaks were observed in all cases at 191, 285, 398, and 532 eV, which corresponded to boron (B 1s), carbon (C 1s), nitrogen (N 1s), and oxygen (O 1s), respectively. Unexpectedly, the C 1s and O 1s peaks also appeared in the pristine hBN (Figure 3a), which might be due to contamination [23]. Meanwhile, the C 1s core-level spectrum of the hBN was decomposed into three peaks that were attributed to C-C (284.8 eV), C-N (285.5 eV), and C-O (286.6 eV) bonds. However, one new peak corresponding to C=O (288.3 eV) bonds emerged in the mhBN, which was derived from the PCPA coating. In comparison with Figure 3b, another additional peak ascribed to O-C=O (289.4 eV) bonds was observed in mhBN@GO (Figure 3c), which was caused by the -COOH groups in GO. This indicated that the GO could be attached to the mhBN surface through the hydrogen bonds between the -COOH groups in GO and the -OH groups in PCPA [24]. 

Moreover, according to Table 1, the C content in the pristine hBN was only 7.79 wt%, whereas that in mhBN dramatically increased to 13.03 wt%, which was due to the deposition of PCPA onto the hBN surface [16]. In addition, the content of O increased from 2.50 wt% for hBN to 5.54 wt% for mhBN@GO, which was attributed to the oxygen-containing groups in GO. These results provided compelling evidence of the successful assembly of the PCPA layer and GO sheets on the hBN surface.

**Figure 4:**
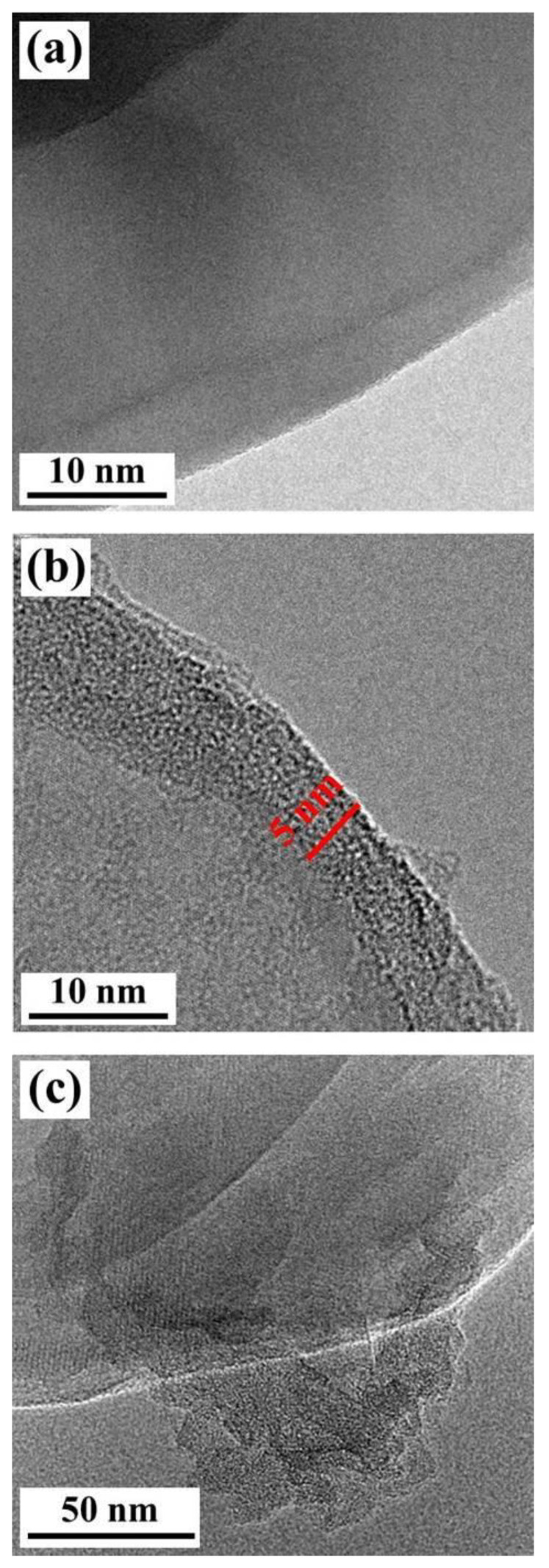
HR-TEM images of (**a**) hBN, (**b**) mhBN, and (**c**) mhBN@GO.

Figure 4 displays the HR-TEM images of the hBN, mhBN, and mhBN@GO. In Figure 4a, the pristine hBN exhibited a homogeneous flat surface without any impurities. Compared with Figure 4a, the surface of the mhBN was coated by a smooth layer with a thickness of approximately 5 nm (Figure 4b), which was due to the formation of a PCPA layer on the hBN surface. As shown in Figure 4c, after the introduction of GO, a large and plicate flake was successfully bonded to the surface of mhBN, indicating strong adhesion of PCPA.

**Figure 5:**
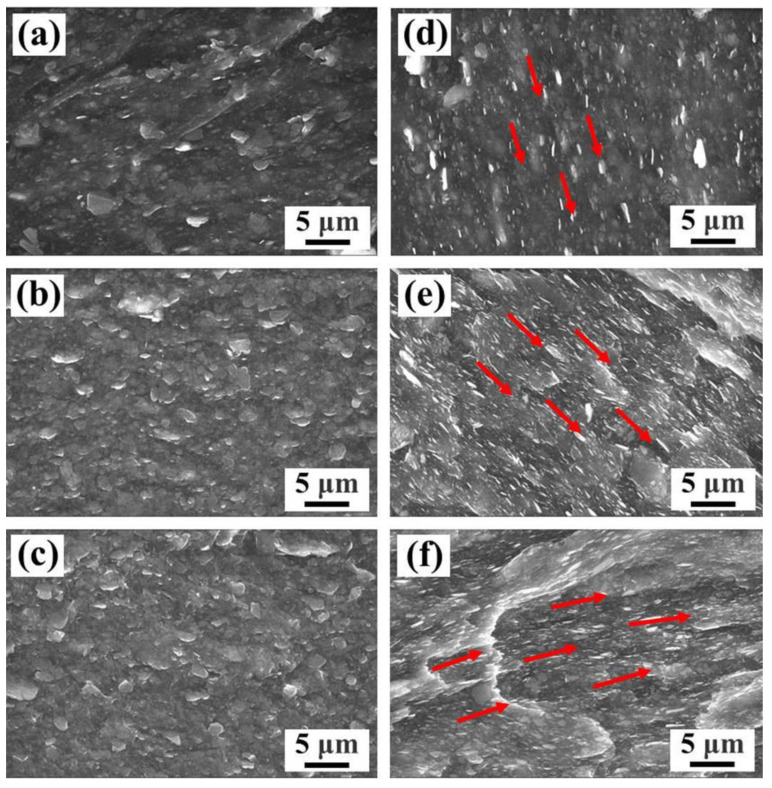
Cross-sectional SEM images of (**a**) 10 vol% hBN/XNBR, (**b**) 20 vol% hBN/XNBR, (**c**) 30 vol% hBN/XNBR, (**d**) 10 vol% mhBN@GO/XNBR, (**e**) 20 vol% mhBN@GO/XNBR, and (**f**) 30 vol% mhBN@GO/XNBR composites. The red arrows represent the orientated dispersion of mhBN@GO.

The cross-sectional SEM images of XNBR composites with different filler contents are shown in Figure 5. As seen from Figure 5a–c, multiple hBN platelets were exposed within the XNBR matrix, indicating their poor compatibility and weak interface interaction. Moreover, the number and size of aggregates between hBN platelets in the XNBR matrix increased with the increase in filler content. Compared to the hBN/XNBR composites, the mhBN@GO hybrids were uniformly dispersed in the XNBR matrix according to certain orientations (see the red arrows in Figure 5d–f). These orientations were beneficial for the formation of heat conduction pathways in the XNBR composites. Furthermore, good compatibility and strong interface bonding between the mhBN@GO hybrids and XNBR matrix would be critical in reducing the interface thermal resistance and phonon scattering of elastomer composites [3,25].

**Figure 6:**
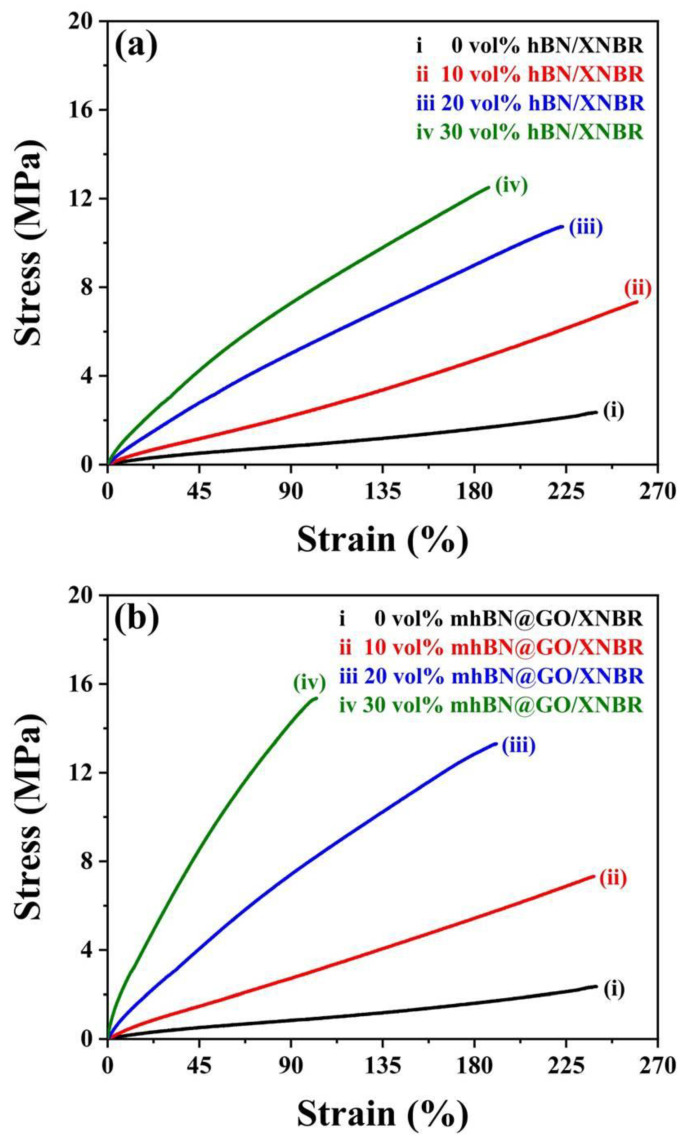
Mechanical properties of (**a**) hBN/XNBR and (**b**) mhBN@GO/XNBR composites.

Figure 6 shows the mechanical properties of XNBR composites filled with various volume fractions of pristine hBN platelets and mhBN@GO hybrids. With the increase in filler content, the tensile strength of both hBN/XNBR and mhBN@GO/XNBR composites increased. However, the tensile strength of mhBN@GO/XNBR composites exceeded that of hBN/XNBR composites at the same filler content. This could be explained by the enforcement effect of mhBN@GO hybrids and the strong interfacial coupling between GO and XNBR [24]. As for the mhBN@GO/XNBR composite with 30 vol% hybrids, the tensile strength increased to 15.35 MPa, which was about 6.50 times that of the neat XNBR (2.36 MPa). Nevertheless, with increasing filler content, the elongation at break of both hBN/XNBR and mhBN@GO/XNBR composites decreased. The mhBN@GO/XNBR composites showed lower elongation at break than the hBN/XNBR composites with the same filler loading, which could be explained by the following two aspects: first, the strong interface interaction between the mhBN@GO hybrids and XNBR matrix might have restricted the slippage of XNBR chains [26]; secondly, owing to the good dispersion and compatibility of mhBN@GO hybrids in XNBR, the filler networks gradually formed in XNBR composites with the increase in mhBN@GO hybrids content [27,28]. The strong interface bonding between the filler and the matrix and the filler networks in polymeric composites usually leads to a lower elongation at break. 

**Figure 7:**
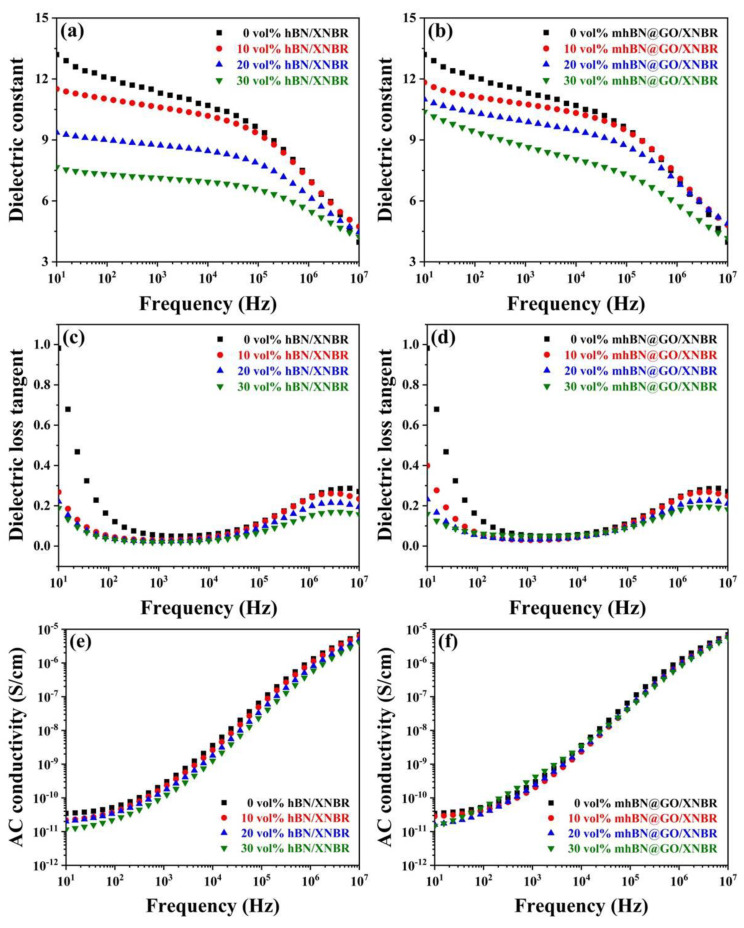
(**a**,**b**) Dielectric constant, (**c**,**d**) dielectric loss tangent, and (**e**,**f**) AC conductivity as functions of frequency for hBN/XNBR and mhBN@GO/XNBR composites, respectively.

Figure 7 displays the frequency dependence of the dielectric behaviors for neat XNBR and XNBR-based composites over the range of 10–10^7^ Hz. In Figure 7a,b, the dielectric constant (*ɛ*_r_) of the XNBR composites decreased gradually with increasing frequency. This suggested that the interface polarization between the filler and the XNBR matrix did not have enough relaxation time to catch up with the change in electric field frequency [29]. Besides, the *ɛ*_r_ of the XNBR composites decreased with the increase of filler content, which was mainly ascribed to the hBN platelets with a *ɛ*_r_ (about 4 at 1 kHz) smaller than that of the neat XNBR (11.30 at 1 kHz) [30]. However, at the same filler content, the mhBN@GO/XNBR composites displayed a higher *ɛ*_r_ than the hBN/XNBR composites (Figure 7a,b). For instance, the *ɛ*_r_ of 30 vol% hBN/XNBR composites decreased to 7.13 at 1 kHz, which was inferior to that of 30 vol% mhBN@GO/XNBR composites (8.64 at 1 kHz). This finding could be explained in terms of the following factors. The first was related to the so-called mini-capacitor principle. With the incorporation of GO, many mini-capacitors with GO as electrodes and XNBR as dielectrics are generated in the composites, leading to a high *ɛ*_r_ [31,32]. Another one was the enhanced interface polarization between the thermally conductive fillers and the XNBR matrix. The interface polarization in the mhBN@GO/XNBR composites occurred at the interfaces between hBN and PCPA, between PCPA and GO, and between GO and XNBR, whereas that in the hBN/XNBR composites was only between the hBN filler and the XNBR matrix [29].

The variation of the dielectric loss tangent (tan*δ*) with frequency in XNBR composites is shown in Figure 7c,d. The tanδ of neat XNBR and XNBR composites first decreases with increasing frequency in the low-frequency range (below 10^3^ Hz) and then increases slightly in the high-frequency region (10^3^–10^7^ Hz). The decreased tanδ in the low-frequency range could be attributed to Maxwell-Wagner-Sillars (MWS) polarization. However, the slightly increased tan*δ* in the high-frequency region is mainly due to dipolar relaxation [29]. It is evident that the tan*δ* of the hBN/XNBR composites has decreased compared to that of the neat XNBR. This was because the hBN acted as an effective insulator to prevent the space charge and leakage current [33]. With increasing content of mhBN@GO hybrids, the tan*δ* of the mhBN@GO/XNBR composites increased from 0.030 to 0.049 at 1 kHz, which was due to the increased leakage current caused by GO [34]. However, the tan*δ* of hBN/XNBR and mhBN@GO/XNBR composites were still maintained at low levels (<0.1 at 1 kHz), indicating that the mhBN effectively hindered the electrical connection between GO [35].

Figure 7e,f depict the frequency-dependent AC conductivity of XNBR composites. According to Maxwell’s equations, the current density j = σ*E and the time derivative of the dielectric displacement dD/dt = iωε*ε_0_E are equivalent, where σ*(ω) is the complex conductivity. Therefore, the conductivity of samples can be calculated according to the following Equations (1) and (2):

(1) $$\sigma *(\omega )=\sigma^{\prime }(\omega )+i\sigma^{\prime \prime }(\omega )=i\omega \varepsilon 0\varepsilon *(\omega )$$

(2) $$\sigma^{\prime }(\omega )=\omega \varepsilon 0\varepsilon^{\prime \prime }(\omega ).\;\sigma^{\prime \prime }(\omega )=\omega \varepsilon 0\varepsilon^{\prime }(\omega )$$

where σ* is the complex conductivity, *σ′* and *σ″* are the real and imaginary parts of the complex conductivity, respectively; ε* is the complex dielectric function or permittivity, *ε′* and *ε″* are the real and imaginary parts of the complex dielectric function, respectively; *ε_0_* is the dielectric permittivity of vacuum (*ε_0_* = 8.854 × 10^–12^ F/m), *i* is imaginary unit *i* = $\sqrt{-1}$, and *ω* is the radial frequency [36]. The AC conductivity of XNBR composites increases gradually with increasing frequency over the range of 10–10^7^ Hz. Obviously, the AC conductivity of the XNBR composites is dependent on the frequency, and it might belong to non-Ohmic conduction [32]. After the addition of hBN or mhBN@GO hybrids, the AC conductivity of the XNBR composites decreased with increasing filler content. The reduction in AC conductivity was mainly due to the lower insulation of the thermally conductive fillers [33]. Notably, the AC conductivity of the XNBR composites was lower than 10^−10^ S/cm below 100 Hz, meaning that the as-prepared mhBN@GO/XNBR composites still maintained excellent electrical insulation [37]. 

**Figure 8:**
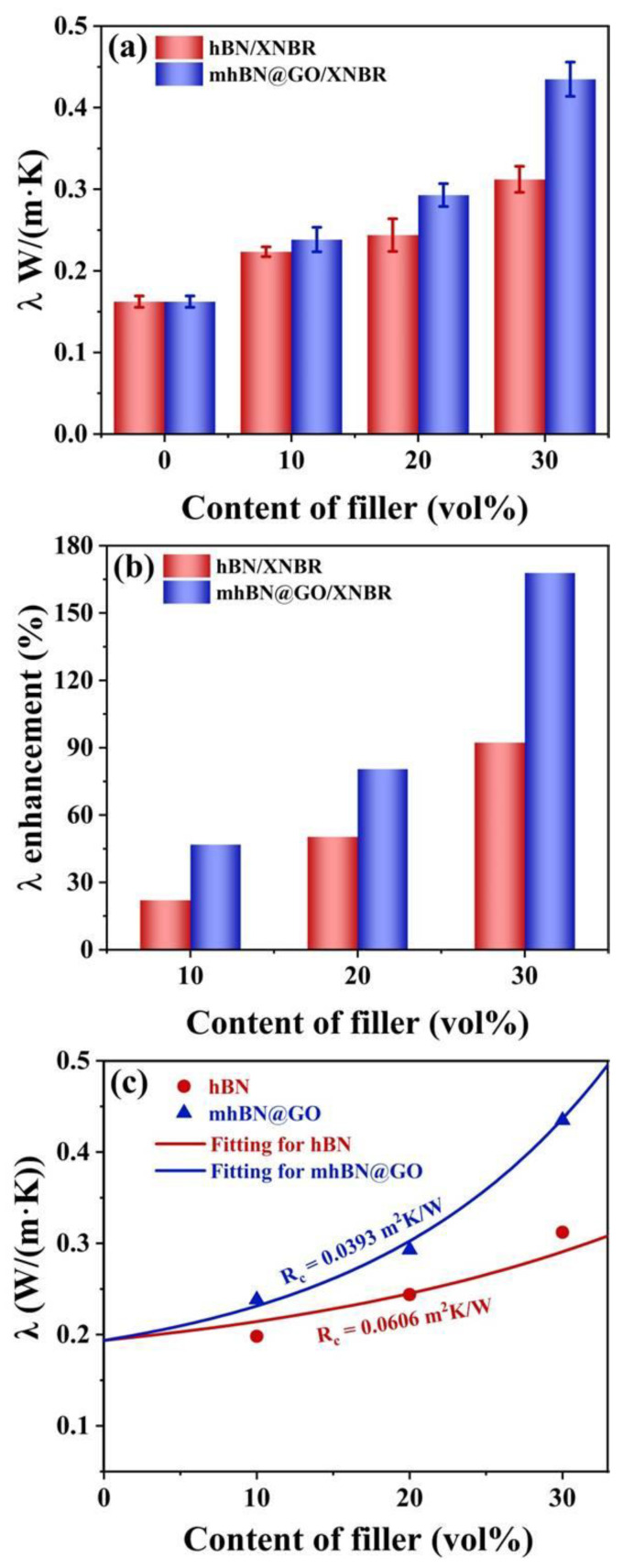
(**a**) The λ of hBN/XNBR and mhBN@GO/XNBR composites with different filler loading. (**b**) The enhancement in λ of hBN/XNBR and mhBN@GO/XNBR composites relative to that of the neat XNBR. (**c**) Fitting λ of hBN/XNBR and mhBN@GO/XNBR composites using the modified Hashin-Shtrikman model.

The λ values of the hBN/XNBR and mhBN@GO/XNBR composites are plotted in Figure 8a. It is worth noting that the λ values of the XNBR composites have been gradually enhanced with increasing additions of filler. Moreover, at the same filler loading, the mhBN@GO/XNBR composites exhibited a higher λ compared to that of the hBN/XNBR composites. As the volume fraction of the mhBN@GO hybrids increased to 30%, the corresponding λ increased to 0.4348 W/(m·K), being about 2.7 times of the neat XNBR (0.1623 W/(m·K)). Figure 8b displays the enhancement in λ of XNBR composites relative to that of the neat XNBR. Compared to the hBN/XNBR composites, a stronger λ enhancement could be observed in the mhBN@GO/XNBR composites. The reasons for this difference were as follows: first, the uniform dispersion of the mhBN@GO hybrids and the construction of continuous heat conduction pathways played an essential role in the heat transfer process within the mhBN@GO/XNBR composites; secondly, due to the low interface thermal resistance, the mhBN@GO/XNBR composites exhibited higher efficiently phonon transmission than the hBN/XNBR composites. 

To expound the internal relationship between the filler and the XNBR matrix, the experimental λ values of XNBR composites (Figure 8c) were fitted using the modified Hashin-Shtrikman model (Equation (3)) [38,39].

(3) $$\lambda_{\mathrm{eff}}=\lambda_{m}\frac{(2KV_{f}+1)\lambda_{c}-(2KV_{f}-1)\lambda_{m}}{(KV_{f}+1)\lambda_{m}-(KV_{f}-1)\lambda_{c}}$$

where λ_m_ is the λ of the XNBR matrix, λ_c_ is the λ of the XNBR composites, V_f_ is the volume fraction of the filler, and K is a coefficient related to the interface thermal resistance of the XNBR composites and defined as Equation (4).

(4) $$K=13.3347\exp(-13.2701\frac{R_{c}\lambda_{m}}{L})$$

where R_c_ stands for the interface thermal resistance and L represents the characteristic length of the unit model [3,40]. According to Figure 8c, the interface thermal resistance of the mhBN@GO/XNBR composites (R_c_ = 0.0393 m^2^K/W) was lower than that of the hBN/XNBR composites (R_c_ = 0.0606 m^2^K/W), demonstrating that PCPA acted as an interface modifier, effectively reducing the interface thermal resistance of XNBR composites. 

**Figure 9:**
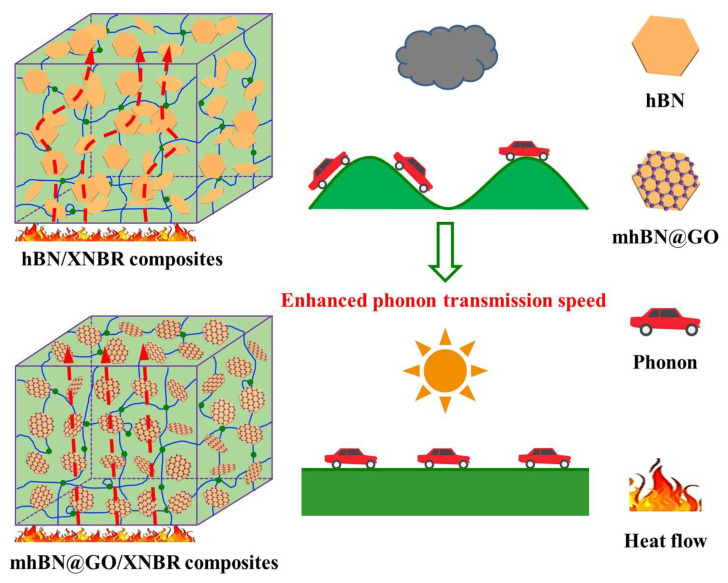
Schematic models of phonon transmission in hBN/XNBR and mhBN@GO/XNBR composites.

Figure 9 displays the schematic models of phonon transmission in the hBN/XNBR and mhBN@GO/XNBR composites. In the hBN/XNBR composites, the hBN platelets are inevitably aggregated in the XNBR matrix, leading to severe scattering of phonons and high interface thermal resistance [41]. In addition, ineffective thermal transfer channels are rendered due to poor dispersion of the hBN in the XNBR matrix, limiting the improvement of λ. As a result, the heat transfer process of the hBN/XNBR composites is like a car moving on a mountain road. However, the heat transfer process of the mhBN@GO/XNBR composites is similar to a car speeding on the highway. Because of the reduced interface thermal resistance, the phonons travel along the continuous heat conduction channels with few barriers. In addition, the ordered heat conduction channels formed by mhBN@GO hybrids make the thermal transfer channels shorter [23].

## 4. Conclusions

The thermally conductive mhBN@GO hybrids were successfully synthesized via a facile and green self-assembly method and then were embedded in XNBR latex to obtain high λ and satisfactory insulating properties of mhBN@GO/XNBR composites. Owing to the formation of hydrogen bonds between carboxyl groups in XNBR and hydroxyl groups in GO, the interfacial interaction between the XNBR matrix and mhBN@GO hybrids was obviously improved, leading to reduced interface thermal resistance and the formation of effective thermal transport pathways in the mhBN@GO/XNBR composites. Thus, the obtained mhBN@GO/XNBR composites exhibited a relatively high λ value of 0.4348 W/(m·K), which was 2.7 times that of the neat XNBR (0.1623 W/(m·K)). However, the insulating hBN effectively prevented the connection between adjacent GO sheets, leading to the mhBN@GO/XNBR composites showing a satisfactory electrical insulating property (less than 10^−10^ S/cm below 100 Hz). Therefore, the effective and eco-friendly method proposed in this work allows one to prepare thermal management materials with insulation properties conforming to the heat dissipation requirements for modern electronic devices.

## Figures and Tables

**Table 1:** Chemical element compositions of hBN, mhBN, and mhBN@GO.

Samples	Elemental Analysis (wt%)			
	B	N	C	O
hBN	50.54	39.17	7.79	2.50
mhBN	46.07	38.07	13.03	2.83
mhBN@GO	40.67	34.48	19.31	5.54

## Data Availability

Not Applicable.
